# Coronavirus disease 2019 (COVID-19) in long-term care facilities: A review of epidemiology, clinical presentations, and containment interventions

**DOI:** 10.1017/ice.2020.1292

**Published:** 2020-10-26

**Authors:** Cameron G. Gmehlin, L. Silvia Munoz-Price

**Affiliations:** Division of Infectious Diseases, Department of Medicine, Medical College of Wisconsin, Milwaukee, WI

## Abstract

Long-term care facilities (LTCFs) and their populations have been greatly affected by the coronavirus disease 2019 (COVID-19) pandemic. In this review, we summarize the literature to describe the current epidemiology of COVID-19 in LTCFs, clinical presentations and outcomes in the LTCF population with COVID-19, containment interventions, and the role of healthcare workers in SARS-CoV-2 transmission in these facilities.

## Background

The severe acute respiratory syndrome coronavirus 2 (SARS-CoV-2) virus was first identified in Wuhan, China, in December 2019 and has since spread widely across the globe; the World Health Organization (WHO) declared it a global pandemic in March of this year.^1,2^ As more patients became ill with the disease, clinicians recognized that coronavirus disease 2019 (COVID-19) disproportionately affects the elderly, in particular long-term care facility (LTCF) residents.^3,4^ As of July 30, 2020, there have been 4,339,997 confirmed cases of COVID-19 and 148,866 associated fatalities in the United States.^5^ Although LTCF residents only make up 3.5% of all COVID-19 cases, this population has contributed to 64.9% of total mortalities due to COVID-19.^6^ A similar situation is occurring in other countries.^7,8^ In this review, we have synthesized the epidemiology of COVID-19 in LTCFs, clinical presentations and outcomes in this population, containment interventions, and the role of LTCF healthcare workers in SARS-CoV-2 transmission.

## Literature review methods

We examined relevant published studies in Medline and PubMed using the following key terms: COVID-19, SARS-CoV-2, assisted living, group home, and memory care. We also reviewed current Centers for Disease Control (CDC) and Centers for Medicare and Medicaid Services (CMS) COVID-19 guidelines for LTCFs.

## Epidemiology

In LTCFs that have COVID-19 cases, SARS-CoV-2 positivity rates can vary widely (Table 1), with an average positivity rate of ~37%. Studies that examined COVID-19 outbreaks in LTCFs had higher attack rates on average (42.9%),^9–18^ but 3 studies surveyed nursing homes without regard to outbreak status and found a SARS-CoV-2 prevalence between 6% and 23%.^19–21^ The highest SARS-CoV-2 positivity rate recorded was by McMichael et al^15^ at 77%, although this was a cumulative attack rate over a month.

**Table 1.** Summary of LTCF Studies Reviewed

Study	No. of LTCFs	Type of LTCF	Clinical Data Presented^a^	Universal Testing for COVID-19	COVID-19 Positivity Rate, %^b^	Hospitalizations, %/Mortality %	Locations
Abrams et al^23^	9,395	SNF	No	No	…	−/−	CA, CO, CT, DE, FL, GA, IA, IL, KY, LA, MA, MD, ME, MI, MN, NC, ND, NJ, NM, NV, NY, OH, OK, OR, RI, SC, TN, VT, WA, WV
Arons et al^9^	1	SNF	Yes	Yes	64	−/26	King County, WA
Blackman et al^30^	1	SNF	No	No	…	−/−	PA
Blain et al^10^	1	SNF	Yes	Yes	48	−/32	France
Borras-Bermejo et al^19^	69	SNF	Yes	Yes	23	−/−	Barcelona, Spain
Dora et al^11^	1	SNF	Yes	Yes	19	89/5	Los Angeles, CA
Escobar et al^12^	1	SNF	Yes	Yes	37	−/−	PA
Feaster et al^13^	9	SNF, ALF	Yes	Yes	70	−/−	Pasadena, CA
Graham et al^14^	4	SNF	Yes	Yes	40	−/17	London, UK
Guery et al^38^	1	SNF	Yes	Yes	…	−/−	France
Harrington et al^26^	272	SNF	No	No	…	−/−	CA
He et al^25^	1,223	SNF	No	No	…	−/−	CA
Li et al^27^	215	SNF	No	No	…	−/−	CT
McMichael et al^15^	1	SNF	Yes	No	77	55/34	King County, WA
Patel et al^16^	1	SNF	Yes	Yes	26	37/29	IL
Roxby et al^18^	1	ALF	Yes	Yes	4	3/1	King County, WA
Rudolph et al^20^	134	SNF	Yes	Yes	6	−/−	United States
Sanchez et al^17^	26	SNF	Yes	Yes	44	37/24	Detroit, MI
Unruh et al^24^	1,162	SNF	No	No	…	−/−	CT, NJ, NY
White et al^21^	3,357^c^	SNF	No	Yes	20	−/−	AL, CA, CO, CT, KY, MA, MD, NH, NJ, NM, NV, PA, RI, TN, VT, WA, WV

Note. LTCF, long-term care facility; SNF, skilled nursing facility; ALF, assisted-living facility; “…” and “–” indicate that data were not available for the corresponding entry.

a Yes = study presents data on prevalence of SARS-CoV-2, hospitalization rates, mortality rates, and/or symptoms of residents and/or staff. No = none of the data are presented.

b Positivity rate encompasses both attack rates and prevalence rates.

c 69 of the 3,357 LTCFs underwent universal testing.

Numerous studies have attempted to identify associations between LTCF characteristics and COVID-19 incidence. Studies using the CMS Five-Star Quality Rating System reported that increased nursing-home size and degree of occupancy appear strongly associated with higher likelihood of having at least 1 resident with COVID-19.^21–24^ LTCF resident demographics also influence the probability of having at least 1 COVID-19 case. Higher proportion of African-American residents, lower proportion of white residents, and higher Medicaid share were all associated with higher numbers of COVID-19 cases.^23–25^ Staffing levels and CMS staffing rating of LTCFs may also affect COVID-19 spread. In California, lower nurse staffing hours per resident per day and lower Five-Star nursing score were both associated with increased risk of COVID-19 cases in the facilities.^26^ Similarly, in Connecticut, decreased nursing hours were associated with an increased number of COVID-19 cases.^27^ Among facilities with at least 1 death attributed to COVID-19, higher numbers of nursing hours were protective.^27^ Other factors associated with greater incidence of COVID-19 included higher levels of LTCF resident independence, higher number of CMS health deficiencies, and for-profit status.^21,23–26^ Li et al found that nurse staffing, CMS Five-Star rating, and concentrations of Medicaid and racial/ethnic minorities in the facilities were associated with COVID-19 in LTCFs that had least 1 case, even after controlling for county-level variables.^27^ White et al^21^ reported that county-level transmission was the strongest predictor of COVID-19 cases in LTCFs across 31 states: for every increase in 1,000 COVID-19 cases per 100,000 residents in any county, there was an associated 33.6% greater likelihood of COVID-19 cases in the facility.^21^

Eight studies evaluated the presence of comorbidities among LTCF resident with COVID-19 (Table 2). Residents with COVID-19 had high rates of hypertension, cardiac disease, diabetes, and cognitive impairment.^9,11,12,15,18^ Comparisons between COVID-19–positive and COVID-19–negative residents showed that renal disease (*P* < .001), pulmonary disease (*P* < .056), dementia (*P* = .023), severe cognitive impairment (*P* < .001), and obesity (*P* = .026) were associated with SARS CoV-2 positivity.^10,14,20^ Furthermore, cardiovascular disease was strongly associated with increased mortality (χ^2^ = 10.8; *P* = .001).^14^

**Table 2.** Comorbidities in LTCF Residents With COVID-19 in Reviewed Studies^a^

Comorbidity^b^	No. (%)
Cancer	2 (3)^12^ 15 (14.9)^15^
Cardiac disease	13 (17)^12^ 102 (23.0)^20^ 2 (50)^18^ 199 (50.5)^14^ 61 (60.4)^15^ 12 (63)^11^ 39 (81)^9^ 32 (84)^10^
Cerebrovascular accident	5 (6.5)^12^ 95 (24.1)^14^ 10 (26)^10^ 19 (40)^9^
Cognitive impairment	16 (21)^12^ 28 (58)^9^ 301 (68.0)^20^ 32 (84)^10^
COPD	8 (10)^12^ 4 (21)^11^
Dementia	223 (56.6)^14^
Diabetes mellitus	15 (19)^12^ 92 (23.4)^14^ 9 (24)^10^ 32 (31.7)^15^ 165 (37.2)^20^ 18 (38)^9^ 2 (50)^18^ 11 (58)^11^
Hypertension	17 (22)^12^ 68 (67.3)^15^ 13 (68)^11^ 309 (69.8)^20^
Immunological	9 (8.9)^15^
Liver disease	6 (5.9)^15^
Obesity	4 (5)^12^ 101 (22.8)^20^ 11 (23)^9^ 1 (25)^18^ 10 (26)^10^ 31 (30.7)^15^ 7 (37)^11^
Pulmonary disease	59 (15)^14^ 9 (24)^10^ 1 (25)^18^ 32 (31.7)^15^ 142 (32)^20^ 18 (38)^9^
Received hemodialysis	1 (1.3)^12^ 3 (6)^9^
Renal disease	3 (4)^12^ 3 (16)^11^ 86 (21.8)^14^ 1 (25)^18^ 18 (38)^9^ 41 (40.6)^15^ 26 (68)^10^

Note. COPD, chronic obstructive pulmonary disease.

a Only studies that preset clinical data are listed.

b Some comorbidity categories were consolidated.

## Clinical presentation

Clinical presentation of COVID-19 can be variable in the LTCF population (Table 3). Four studies reported that between 69.7% and 93% of LTCF residents experienced typical COVID-19 symptoms such as fever, cough, dyspnea, or hypoxia.^9,10,15,19^ Other case reports have shown a high prevalence of fevers and cough alongside anorexia, headaches, diarrhea, and fatigue.^11,16^ Graham et al^14^ reported a strong association between anorexia, cough, and breathlessness and SARS-CoV-2 positivity. Given the low rate of fever among the elderly, it was not surprising that fever was not associated with increased odds of having a positive SARS-CoV-2 test. In fact, a study by Rudolph et al^20^ showed that only 26.6% of SARS-CoV-2 positive residents reached a fever of 38.0°C during their 28-day observation period. Neither anosmia or ageusia have been reported in the literature among this population.

**Table 3.** Signs and Symptoms Among COVID-19 LTCF Residents in Reviewed Studies^a^

Symptoms^b^	No. (%)
**Typical** ^c^	211 (30.7)^19^ 17 (80.9)^9^
Fever	118 (26.6)^20^ 211 (30.7)^19d^ 30 (41.7)^14^ 15 (43)^16^ 27 (74)^10^ 11 (84.6)^11^
Cough	1 (25)^18^ 9 (26)^16^ 211 (30.7)^19d^ 14 (37)^10^ 7 (53.9)^11^ 41 (59.9)^14e^ 111 (-)^15e^
Dyspnea	5 (14)^16^ 3 (23.1)^11^ 211 (30.7)^19d^ 41 (59.9)^14e^ 24 (63)^10^ 111 (-)^15e^
Hypoxia	1 (3)^16^ 21 (55)^10^
**Atypical** ^c^	6 (16)^10^ 4 (19)^9^
Chills	1 (3)^16^
Malaise	9 (26)^16^
Sore throat	3 (9)^16^
Confusion	43 (59.7)^14^
Rhinorrhea	…
Nasal congestion	…
Myalgia	2 (6)^16^ 1 (7.7)^11^
Dizziness	…
Headache	2 (15.4)^11^
Nausea/Vomiting	2 (15.4)^11^
Diarrhea	2 (2.8)^14^
Decreased appetite	4 (11)^16^ 4 (30.8)^11^ 34 (47.2)^14^
Seizures	1 (3)^16^
LOC	1 (3)^16^

Note. LTCF, long-term care facility; LOC, loss of consciousness; “…” indicates that data was not available for the corresponding entry.

a Only studies that present clinical data are listed.

b Some symptom categories were consolidated.

c Categories of “typical” and “atypical” are based on the CDC symptom categorization.

d This study reported an aggregate incidence rate of fever, cough, and dyspnea.

e These studies reported aggregate incidence rates of cough and dyspnea.

Interestingly, typical COVID-19 symptoms were observed in SARS-CoV-2–negative residents, ranging from 10.6% to 54%.^9,10,14,18,19^ Asymptomatic SARS-CoV-2 infections were also identified, with rates from 16% to 69.7%.^9,10,12-14,16,17,19^ In studies by Arons et al^9^ and Escobar et al,^12^ 88%–92% of residents with asymptomatic infections developed symptoms during subsequent follow up. By contrast, Graham et al^14^ and Patel et al^16^ reported only 3% to 10% of asymptomatic infections developing subsequent symptoms among nursing-home residents.

## Prognosis

Following COVID-19 diagnosis, many LTCF residents required subsequent hospitalization or expired (Table 1). The average hospitalization rate of SARS-CoV-2–positive residents across all studies was 44% and the average mortality rate was 21%.^9-11,14–18^ The highest hospitalization rate was reported by Dora et al^11^ at 89%; however, these researchers stated that transfers to acute-care settings were primarily driven early in the pandemic by the need to isolate these residents from others in the facility. The highest fatality rate reported to date was 33.7% in a 130-bed LTCF in Washington State.^15^

## Containment interventions

A wide array of interventions have been used by LTCFs to prevent and/or halt outbreaks of COVID-19 (Table 4). Surveillance and social distancing were used almost universally, with group activities cancelled and daily screening of residents, staff, and visitors effected.^9,15^ Visitation restrictions were also enacted upon recommendation of the CDC and CMS to prevent introduction of COVID-19 from the community into LTCFs.^28,29^ Although no studies have examined this issue directly, reports by Graham et al^14^ and Blackman et al^30^ show that, even with stringent visitation restrictions, COVID-19 can still be introduced to LTCFs. Once an outbreak occurred, cohorting was universally used in an attempt to mitigate the spread of COVID-19. However, due to limited supplies of SARS-CoV-2 rRT-PCR tests, universal testing among residents and staff was not performed, thereby limiting the effectiveness of cohorting interventions.^30,31^ As tests became more widely available and evidence emerged of asymptomatic transmission, universal testing during LTCF outbreaks was recommended.^9,32^ Cohorting and universal testing have proven effective; facilities in California and Illinois that utilized these methods saw a significant reduction in incidence and fatality rate when compared to earlier studies that did not use universal testing to guide cohorting.^9,11,15,16,30^ In May 2020, the CDC recommended that all nursing-home residents and healthcare workers (HCWs) should be tested if a case of COVID-19 is detected, followed by weekly testing of negative residents until no new cases are detected.^33^ Subsequent studies have reinforced the value of this approach.^10,12,17^

**Table 4.** Containment Interventions Utilized by LTCF in Reviewed Studies^a^

Study	Before Outbreak	After Outbreak
Arons et al^9^	Daily resident symptom assessment, daily staff symptom assessment	Point prevalence testing of residents, point prevalence testing of staff, restriction of visitation,^b^ social distancing,^c^ universal transmission-based precautions, universal use of PPE
Blackman et al^30^	Daily resident symptom assessment, daily staff symptom assessment, infection control training, restriction of visitation,^b^ social distancing^c^	Cohorting of residents and staff, symptom-based testing of residents, universal transmission-based precautions, universal use of PPE
Blain et al^10^	…	Point prevalence testing of residents, point prevalence testing of staff
Borras-Bermejo et al^19^	Restriction of visitation^b^	Cohorting of residents and staff, infection control training, point prevalence testing of residents, point prevalence testing of staff, social distancing^d^
Dora et al^11^	Daily resident symptom assessment, daily staff symptom assessment, restriction of visitation^b^	Cohorting of residents and staff, creation of COVID-19 ward, infection control training, point prevalence testing of staff, serial testing of residents, social distancing^c^
Escobar et al^12^	Daily resident symptom assessment, daily staff symptom assessment, point prevalence testing of staff, restriction of visitation,^b^ social distancing,^c^ suspension of admissions, use of metered inhalers vs nebulizers	Cohorting of residents and staff, creation of COVID-19 ward, serial testing of residents, universal use of PPE
Feaster et al^13^	…	Cohorting of residents and staff, point prevalence testing of residents, point prevalence testing of staff
Graham et al^14^	…	Point prevalence testing of residents, convenience testing of staff
McMichael et al^15^	…	Infection control training, symptom-based testing of residents
Patel et al^16^	…	Cohorting of residents and staff, daily staff symptom assessment, infection control training, point prevalence testing of residents, point prevalence testing of staff, restriction of visitors,^b^ universal masking of residents, universal use of PPE
Roxby et al^18^	…	Daily staff symptom assessment, point prevalence testing of residents, point prevalence testing of staff, social distancing^c^
Sanchez et al^17^	Symptom-based testing of residents	Cohorting of residents and staff, creation of COVID-19 ward, infection control training, point prevalence testing of staff, serial testing of residents

Note. LTCF, long-term care facility; PPE, personal protective equipment; “…” indicates that a containment intervention was not listed for the corresponding period.

a Only studies describing containment interventions are listed.

b All visitors restricted from entering the facility.

c Communal dining and activities cancelled.

d Communal dining and activities cancelled but residents continued to share rooms.

## Role of HCWs

Nosocomial transmission in medical settings is often driven by HCWs unknowingly transmitting illnesses to the patients they care for.^34^ Given that HCWs have been found to be asymptomatic carriers of SARS-CoV-2, current recommendations stress the use of personal protective equipment to prevent viral spread within LTCFs.^28,35,36^ Three studies presented epidemiological evidence of such transmission. In a Pennsylvania LTCF, 2 HCWs who lived together but worked on different units concurrently tested positive for SARS-CoV-2, causing clusters of cases on their respective units.^30^ In King County, Washington, interfacility spread of COVID-19 was facilitated via shared HCWs who worked at multiple facilities.^15^ In addition, genetic sequencing data collected during an outbreak in a London LTCF showed similar SARS-CoV-2 sequence data among a group of residents and a single HCW who cared for them.^14^

A prevalence study of SARS-CoV-2 infection among general practitioners and nurses from primary-care centers and nursing homes in León, Spain, reported that the prevalence of SARS-CoV-2 was higher in nursing homes than in primary-care centers (9.5% vs 5.5%). However, no statistically significant differences were observed by sex, type of professional, level of exposure, or compliance with preventative measures.^37^ In other studies that measured COVID-19 prevalence among LTCF HCWs, values ranged from 2.2% to 62.6%.^9-13,15,16,18,19,38^ Positive SARS-CoV-2 cases in LTCF studies included both frontline nursing staff as well as ancillary workers. Although most SARS-CoV-2–positive cases have occurred among LTCF nursing staff, other personnel, such as physicians, physical, speech and occupational therapists, case managers, health information officers, and environmental services, have also been affected.^9,15^

Identification of positive HCWs via symptom screening can be problematic because HCWs in LTCFs can be asymptomatic, with only 19% to 55.8% of staff exhibiting symptoms such as cough, fever, sore throat, dyspnea, headaches, or myalgias.^9,16,18,19^ Further complicating infection prevention measures, 9.75% to 40% of HCWs with negative SARS-CoV-2 test results had symptoms characteristic of COVID-19, and 4% to 55.8% were asymptomatic with a positive test.^9,10,13,14,18,19,37,38^

Many COVID-19 infection prevention measures are focused on staff use of personal protective equipment (PPE) and, therefore, can be hindered by shortages of these supplies. In a survey of US LTCFs early in the pandemic, 72% reported having inadequate access to PPE, 88% of surveyed facilities reporting a shortage of face shields, and 64% of surveyed facilities reporting a shortage of surgical masks.^39^ HCWs in LTCFs have continued to work despite having symptoms consistent with COVID-19. In a study of 50 HCWs who tested positive during the initial cluster of COVID-19 in Washington State, 64% reported working while exhibiting symptoms.^40^

In conclusion, our review of recent studies and guidelines for COVID-19 in LTCFs has identified several key observations as well as areas for further investigation. First, emerging data indicate that certain facility characteristics are associated with increased likelihood of having at least 1 COVID-19 case in an LTCF: Five-Star rating, resident demographics, staffing levels, and county-level transmission. In addition, once SARS-CoV-2 is introduced into an LTCF, it can quickly spread, leading to high rates of morbidity, hospitalization, and mortality. Infection control interventions, such as cohorting and universal testing of staff and residents, appear to be effective. Many studies have indicated the effectiveness of these strategies to mitigate COVID-19 outbreaks. Given the disproportionate transmission, morbidity, and mortality in the nursing-home population, more studies are needed that incorporate novel containment interventions in LTCFs.
